# Reptile biodiversity in Souss-Massa National Park: an internationally important hotspot in the Mediterranean region

**DOI:** 10.3897/BDJ.10.e79088

**Published:** 2022-02-16

**Authors:** Abderrafea Elbahi, Colin Lawton, Widade Oubrou, Mohammed El Bekkay, Jamila Hermas, Michel Dugon

**Affiliations:** 1 Venom Systems and Proteomics Lab, Ryan Institute, National University of Ireland, Galway, Ireland Venom Systems and Proteomics Lab, Ryan Institute, National University of Ireland Galway Ireland; 2 Laboratory of Aquatic Systems: Marine and Continental Environments (AQUAMAR), Faculty of Sciences, Ibn Zohr University, Agadir, Morocco Laboratory of Aquatic Systems: Marine and Continental Environments (AQUAMAR), Faculty of Sciences, Ibn Zohr University Agadir Morocco; 3 Zoology, School of Natural Sciences, Ryan Institute, National University of Ireland, Galway, Ireland Zoology, School of Natural Sciences, Ryan Institute, National University of Ireland Galway Ireland; 4 Souss-Massa National Park, BP. 107-Inezgane, Inezgane, Morocco Souss-Massa National Park, BP. 107-Inezgane Inezgane Morocco

**Keywords:** accumulation curves, inventory, non-parametric estimators, species richness, time-constrained visual encounter surveys

## Abstract

Souss-Massa National Park (SMNP) is Morocco’s first coastal national park, created to preserve the high diversity of its continental and marine environments. Reptiles play an essential role in balancing SMNP ecosystems, yet little work has been done to study this fauna. The present work aims at providing the first reptile inventory of SMNP since its establishment in 1991. During the period 2019 to 2020, several field surveys were carried out at 30 sites using time-constrained visual encounter surveys (TCVES), with a total sampling effort of 300 person-hours. An inventory of 23 reptile species (including four endemic species) was obtained by combining TCVES results with additional data recorded during random encounters or provided by SMNP researchers. Based on TCVES data, both sampling effort and inventory completeness were evaluated by constructing sample-based accumulation curves and calculating non-parametric estimators (Chao 1, Chao 2, Jackknife 1 and Jackknife 2). These species richness estimators suggest that the current inventory is likely to be complete. Despite its small surface area, SMNP contains nearly 20% of all known Moroccan reptile species and constitutes an important biodiversity hotspot for reptiles in the Mediterranean Region. In terms of reptile conservation concern, five species in SMNP are classified as “vulnerable”, while two species are classified as “near threatened” on the IUCN Red List, underscoring the importance of protected areas for those species.

## Introduction

The Mediterranean Basin is the world's second largest biodiversity hotspot (Mittermeier et al. 2004). Over the last few decades, overexploitation of natural resources, habitat loss and pollution due to direct and indirect human activities have caused severe threats to the Mediterranean species (Cuttelod et al. 2008). With the continued loss of biodiversity and habitat destruction occurring in the Mediterranean Region, several conservation strategies have been developed to maintain biodiversity and preserve natural habitats and ecosystem processes. These strategies rely primarily on protected areas, which are defined as recognised geographical spaces created to support the long-term conservation of the environment and wildlife, as well as ecosystem services and cultural values. These spaces are managed through legal or other effective means (Dudley 2008).

Morocco was ranked as the second richest country in term of biodiversity in the Mediterranean Basin after Turkey (MATEE 2009). This North African country has the richest and the most varied herpetofauna of the western Mediterranean and the Maghreb Region (de Pous et al. 2010). This diverse fauna is characterised by a high rate of endemism and relict species. This is due to several factors: a high diversity of habitats, a wide range of climate types and the presence of several geographic barriers (such as the Atlas and the Rif Mountains), which can lead to allopatric speciation (Schleich et al. 1996, Brown et al. 2002, Lehr et al. 2005, Fritz et al. 2006, Kane et al. 2019, Martínez del Mármol et al. 2019). In order to protect this high diversity of species and their habitats, the Moroccan Department of Water and Forests contributed to the creation of several terrestrial and marine protected areas, such as national parks, biosphere reserves, natural reserves and Sites of Biological and Ecological Interest (SBEI).

National parks were one of the first established protected areas in Morocco. They are spread through the country and host key habitats for endemic, threatened and emblematic species, as well as their associated ecosystems. Historically, Souss-Massa National Park (SMNP) became Morocco’s first coastal national park when it was established in 1991, with the main purpose of conserving its diverse continental and marine environments. Alongside the Atlantic coastal strip of the Souss-Massa Region, SMNP is qualified as a biodiversity hotspot area within the Mediterranean Basin (Mittermeier et al. 2004). It is considered the heart of the first biosphere reserve in Morocco, known as the Arganeraie Biosphere Reserve (UNESCO 2002), which hosts the endemic argan tree (*Arganiaspinosa*), a species in need of conservation. SMNP takes its name from the Souss and Massa Rivers that flow through its territory. In 2005, both Souss and Massa River mouths were added to the “Ramsar List of Wetlands of International Importance” (Ramsar Convention 2019), as they are important sites for endemic and migratory birds. This National Park is one of two remaining areas that host the world’s last northern bald ibis (*Geronticuseremita*) (Aourir et al. 2017).

So far, very little work has been done to study SMNP reptiles and available data remain very insufficient. Early literature shows that the first most important reptile records in the Souss-Massa Region were made by Bons (1959) and Stemmler (1972). These records were then followed by a herpetological inventory of what was previously called Massa National Park Project (MNPP), which was supposed to cover 48,000 hectares compared to 33,800 hectares for the actual National Park. This herpetological inventory was published by Mellado et al. (1988) (three years before the creation of SMNP) and it was included in their work on amphibian and reptile ecology of MNPP. Thirty years after establishing SMNP, no further herpetological inventories have been made and data on its reptilian fauna have been limited to scattered records, mostly in its vicinity. These records were mainly made by Bons and Geniez (1996) and Martínez del Mármol et al. (2019). The current work aims to provide the first reptile inventory of SMNP since its establishment in 1991 and to compare this inventory with the previous one made by Mellado et al. (1988), during the preparatory phase of MNPP. This work reports the findings from time-constrained visual encounter surveys (TCVES) and presents reptile species records’ results combined with additional data recorded during random encounters or provided by SMNP researchers. Based on TCVES technique data, both sampling effort and inventory completeness were evaluated by constructing sample-based accumulation curves and calculating non-parametric estimators.

## Materials and Methods

### Study area

**Figure 1.**
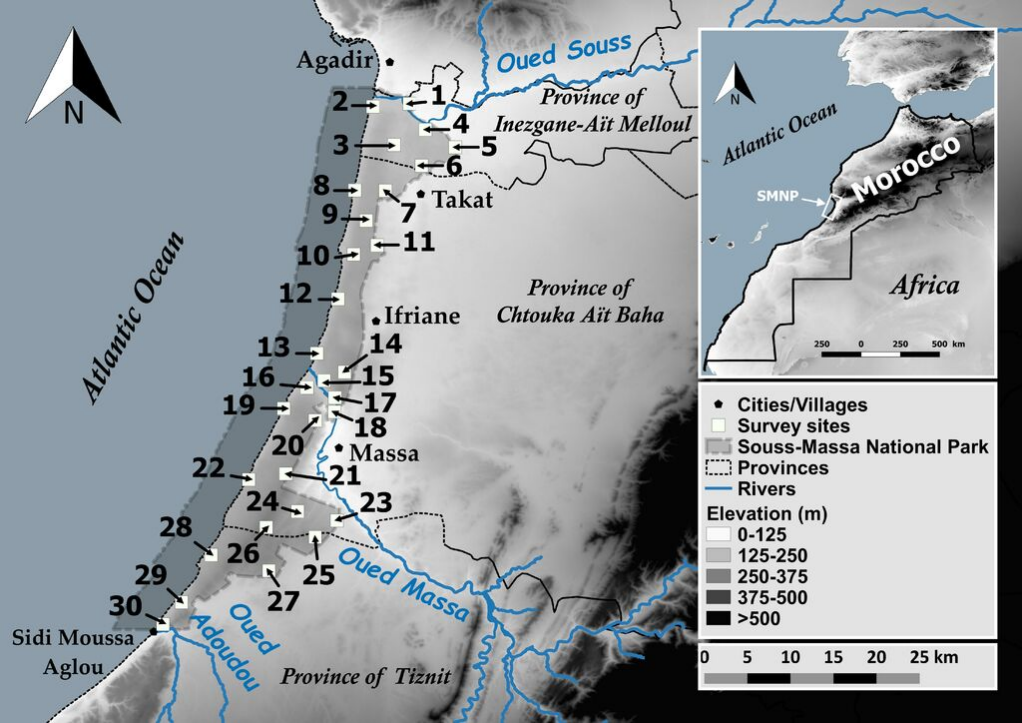
Map showing the surveyed sites within Souss-Massa National Park.

The study was conducted at SMNP, located along the Moroccan Atlantic coast of the Souss-Massa Region and administratively part of three provinces: Inezgane-Ait Melloul, Chtouka Ait Baha and Tiznit. This National Park covers 33,800 hectares and includes two main zones; 12,350 hectares of forest reserves and 21,450 hectares of private and collective lands. SMNP stretches over no less than 65 km, from the Souss River mouth in the north to the Adoudou River mouth near Sidi Moussa Aglou in the south (Fig. 1). Its centre is located at the Massa River mouth. SMNP consists of a long low-lying narrow strip (5 km wide in average) of plains and hills situated between the High Atlas and the Anti-Atlas Mountains, with an altitude ranging between 0 and 200 m. Most of its areas consist of cliffs, coastal consolidated and migrating sand dunes, as well as calcareous rocky steppes.

SMNP has a mean annual temperature of 18°–20°C and an annual average precipitation that varies between 100 and 300 mm (Mokhtari et al. 2014, Climate-Data.org 2019). Its vegetation community belongs to the “Mediterranean Acacia-Argania dry woodlands and succulent thickets” ecoregion and is categorised within the “Mediterranean Forests, Woodlands & Scrub” biome (Olson et al. 2001). This National Park’s natural vegetation is very diverse and comprises several formations, mainly: coastal steppe distributed on sandy soils and dominated mainly by *Retamamonosperma*, *Launaeaarborescens*, *Ononisnatrix* and *Asteriscusimbricatus*. Followed by *Euphorbia* steppe, dominated by *Euphorbiaechinus*, *Euphorbiabeaumierana* and *Euphorbiaregis-jubae*. The endemic argan woodlands is confined to the northeast of the National Park. Both Souss and Massa Rivers' wetlands are characterised by halophytes and hydrophytes. Some natural habitats have been modified (in order to stabilise sand dunes) by planting *Eucalyptus* (non-native) and *Acacia* trees, especially in the northern part of the National Park. Other habitats have been cleared for farming.

### Data collection

From 8 January 2019 to 5 December 2020, several field surveys were carried out inside SMNP, focusing on determining reptile species richness. Thirty different sites (Fig. 1) were selected and explored, based on initial reconnaissance surveys, habitat structure and the possibility of reptile species availability. The time-constrained visual encounter surveys (TCVES) were used to standardise sampling efforts. This technique involves an active visual search for species in a given area for a pre-defined amount of time (Eekhout 2010, McDiarmid et al. 2012). Both diurnal and nocturnal surveys were carried out, with a total sampling effort of 300 person-hours (the entire sampling and observations were made by the same observer, A. Elbahi). Diurnal surveys were conducted once per season at each site for a duration of three hours (between 09:00 h and 17:00 h), except for wildlife enclosures, site 26 and site 28, which were only surveyed once during the whole study period due to their limited or difficult access. In addition, nocturnal surveys were performed once at sites near villages for a duration of an hour and a half (between 20:00 h and 23:00 h) using flashlights or headlights.

We tried to cover several microhabitats commonly occupied by reptiles from sea level to the top of hills during the active search. We searched surfaces and vegetation, turned over objects, such as logs and rocks and looked in crevices in rocks, replacing all surface objects after examining the ground beneath. We looked for active reptiles and those hiding beneath rocks and explored water bodies for aquatic reptile species. Furthermore, we determined the presence of some reptiles based on their faecal droppings, tracks, carcasses and shed skin (particularly those of snakes and amphisbaenians). Sampling inside enclosures was mainly restricted to the areas along trails to avoid disturbing protected antelopes and ostriches. All reptile species were recorded, as well as their GPS (Global Positioning System) coordinates, elevation and date. We then combined our survey data with some Additional Observations (AO) recorded during random encounters or provided by SMNP researchers. All AO entries were supported by photo-vouchers as reliable evidence of presence in the study area.

Several specimens were captured (by hand or with a snake hook) under a scientific permit number No. 20-2019, issued by the “High Commission for Water and Forests and the Fight against Desertification” (HCEFLCD), in addition to decision No. 17/19 and an internship agreement provided by SMNP authorities. For each capture, the snout-vent length (SVL) was measured using Vernier calipers (± 0.1 mm), as well as weight, sex and age class (juvenile and adult). Each individual was then released alive in its natural habitat. The time taken for species identification and data collection was not included in the total time-constrained searches. Terrestrial reptile identification was done, based on scalation and morphometrics using field guides (Bons and Geniez 1996, Schleich et al. 1996, Martínez del Mármol et al. 2019). Sea turtles identification was made, based on the work of Pritchard and Mortimer (1999). Dead specimens were collected from the wild and stored at the Faculty of Science, University Ibn Zohr, Agadir, Morocco.

### Data analysis

In order to evaluate sampling effort and inventory completeness using the TCVES technique, we constructed sample-based accumulation curves. The non-parametric estimators Chao 1, Chao 2, Jackknife 1 and Jackknife 2 were used to estimate total species richness. These estimators were chosen, based on previous studies supported by the work of Walther and Martin (2008), which confirms that, for their dataset, the two Chao estimators were generally the most precise estimation methods and the least biased, followed by the two Jackknife estimators. EstimateS version 9.1.0 software (Colwell 2013) was used to calculate abundance-based (Chao 1) and incidence-based estimators (Chao 2, Jackknife 1 and Jackknife 2), in addition to the species accumulation curves. We performed 100 randomisation runs without replacement and used the bias-corrected form of the Chao richness estimators. Inventory completeness was measured as the percentage that the observed species richness represented of the total estimated species richness.

## Results

We obtained an inventory comprising 23 species spanning across 15 families and 19 genera (as shown in Table 1). This includes four species of Chelonians and 19 species of Squamates. Amongst Chelonians, all families (Testudinidae, Geoemydidae, Cheloniidae and Dermochelyidae) were represented by one species each. Amongst Squamates, the Scincidae family was the most diverse with four species, followed by the Lacertidae family, which contained three species. The families Phyllodactylidae, Colubridae and Lamprophiidae were represented by two species each, while the families Sphaerodactylidae, Trogonophiidae, Agamidae, Chamaeleonidae, Elapidae and Viperidae were each represented by a single species. Inventory completeness and species accounts are provided below.

### Inventory completeness

**Figure 2.**
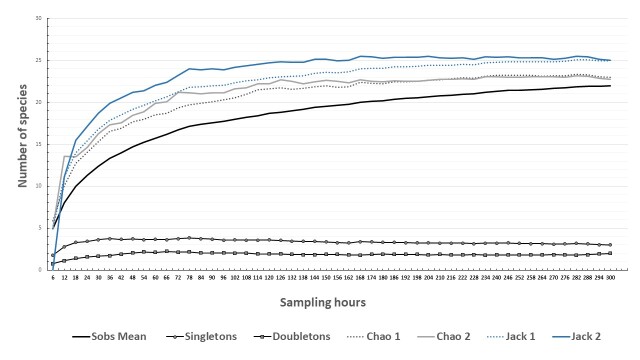
Species accumulation curves for reptiles inside Souss-Massa National Park. Observed richness (Sobs), species represented by a single individual (singletons), species represented by two individuals (doubletons) and estimated species (Chao 1, Chao 2, Jackknife 1 and Jackknife 2).

The observed species accumulation curve appeared to be nearing an asymptote at 22 species (Fig. 2), indicating that the sampling using the TCVES was effective. The non-parametric estimators predicted a total richness of 23 ± 1.82 (Chao1 ± SD), 22.74 ± 1.4 (Chao 2 ± SD), 24.94 ± 1.66 (Jackknife 1 ± SD) and 25 species (Jackknife 2, no SD available), with an inventory completeness of 95.65%, 96.74%, 88.21% and 88%, respectively. The curves of singletons and doubletons for the entire inventory did not intersect; three species (13.63%) were singletons, while two species (9.1%) were doubletons. Overall, the sampling effort using the TCVES was effective because both Chao and Jackknife estimators’ accumulation curves seemed to reach their asymptote.

### Species accounts

**Order Chelonii**

**Family Testudinidae**

**Figure 3.**
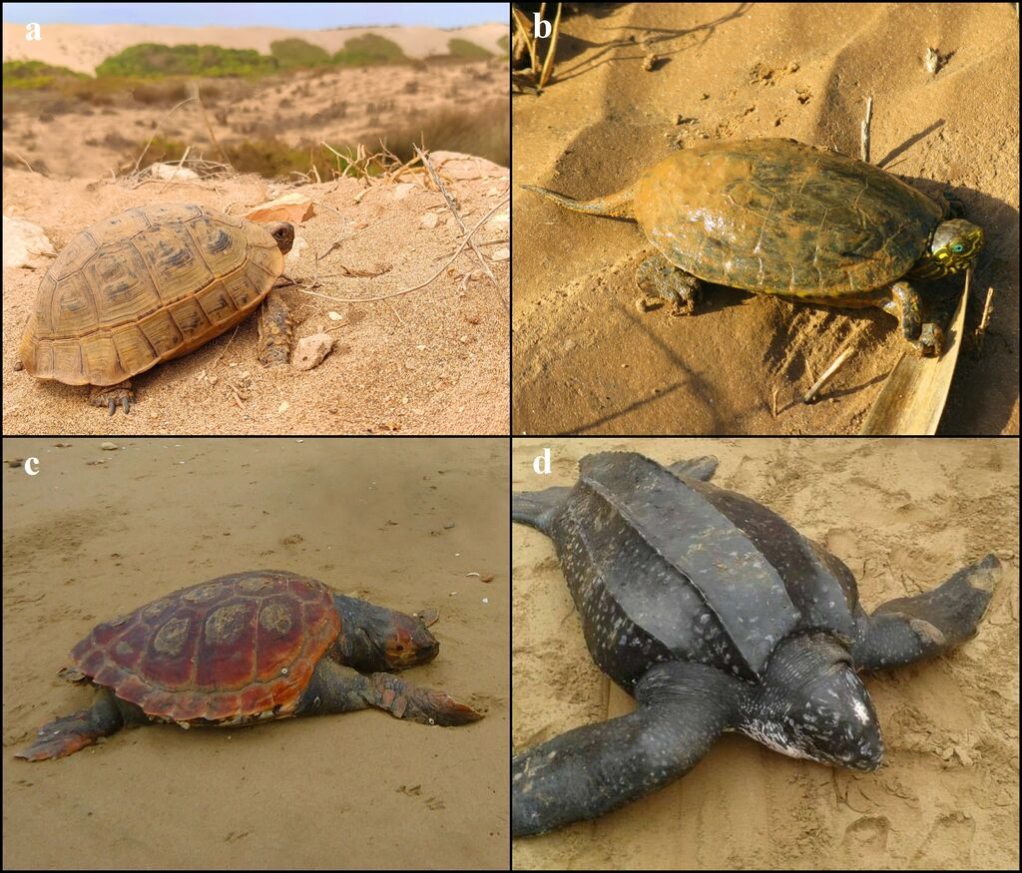
Species of Chelonians present in Souss-Massa National Park. **a** *Testudograeca*, photo by A. Elbahi; **b** *Mauremysleprosa*, photo by W. Oubrou; **c** *Carettacaretta*, photo by M. Iazza; **d** *Dermochelyscoriacea*, photo by M. Bargache.

*Testudograeca* (Fig. 3a)

**Common name**: Spur-thighed Tortoise

**IUCN status**: Vulnerable

**Comment**: It is the only species of tortoise in Morocco and is represented by three subspecies (Fritz et al. 2009):*T.g.soussensis*, *T.g.marokkensis* (both are endemic to the country) and *T.g.graeca*, which is distributed between eastern Morocco, northern Algeria and southern Spain. This terrestrial tortoise is represented in SMNP by the *T.g.soussensis* subspecies that was found at 26 sites (which represents 86.66% of all surveyed sites) with the widest distribution compared to all reptile species in the study area. Sexual dimorphism has been observed amongst this species. Generally, females grow larger than males and are characterised by a flat plastron, while males have a concave plastron.

**Family Geoemydidae**

*Mauremysleprosa* (Fig. 3b)

**Common name**: Mediterranean pond turtle

**IUCN status**: Vulnerable

**Comment**: *Mauremysleprosa* is one of the two aquatic terrapin species that can be found in Morocco. Based on genetic data (Fritz et al. 2006), two subspecies are present in the country; *M.l.leprosa*, which occurs north of the Atlas Mountains and *M.l.saharica*, distributed in the southern (including SMNP) and eastern parts of the country (Bertolero and Busack 2017). These subspecies are separated by the Atlas Mountains with a large contact zone located in the Rif and Middle Atlas Mountains (Veríssimo et al. 2016). During the study period, this freshwater terrapin appeared to be confined to site 18, where it was sighted basking or swimming in the Massa River. They flee instantly and dive into water at the slightest disturbance. All observed individuals had a blue iris, which can be seen in other populations of the *M.l.saharica* subspecies. As was the case with *T.graeca*, sexual dimorphism in *M.leprosa* is remarkable, with females growing larger and having a higher shell with flat plastron, unlike males that have a concave plastron.

**Family Cheloniidae**

*Carettacaretta* (Fig. 3c)

**Common name**: Loggerhead Sea Turtle

**IUCN status**: Vulnerable

**Comment**: A hard‐shelled sea turtle, widely distributed in temperate, subtropical and tropical waters of the Atlantic, Pacific and Indian Oceans (Dodd 1988). Adults and subadults of this species are characterised by a carapace covered with large scales with a uniform reddish-brown colour. They can be distinguished by a carapace broadest anteriorly with a narrower posterior, a “hump” at the fifth vertebral scute and a very large head (Pritchard and Mortimer 1999). *C.caretta* carcasses and carapace fragments were found at five sites (2, 8, 13, 19 and 22) along the SMNP coast. These are the first documented records of this species in the National Park.

**Family Dermochelyidae**

*Dermochelyscoriacea* (Fig. 3d)

**Common name**: Leatherback Sea Turtle

**IUCN status**: Vulnerable

**Comment**: This cosmopolitan reptile is considered the largest of all living turtles and the only living representative of the family Dermochelyidae. It also has the widest distribution range of all reptiles (Bons and Geniez 1996). Adults and subadults of *D.coriacea* can easily be distinguished from other sea turtles by their soft leathery scuteless carapace (soft-shelled) with seven prominent longitudinal keels (ridges) (Pritchard and Mortimer 1999), instead of the hard bony shell with scales found in hard‐shelled sea turtles. Unlike adults and subadults, the hatchlings are characterised by a carapace covered with small, soft, polygonal scales (Pritchard and Mortimer 1999). In SMNP, all *D.coriacea* individuals were recorded as being stranded at one of three sites; site 8, site 13 and site 30. All these observations took place during the year 2015 and were obtained from the AO database. However, no individuals were found stranded during the sampling period 2019-2020.

**Order Squamata**

**Family Sphaerodactylidae**

**Figure 4.**
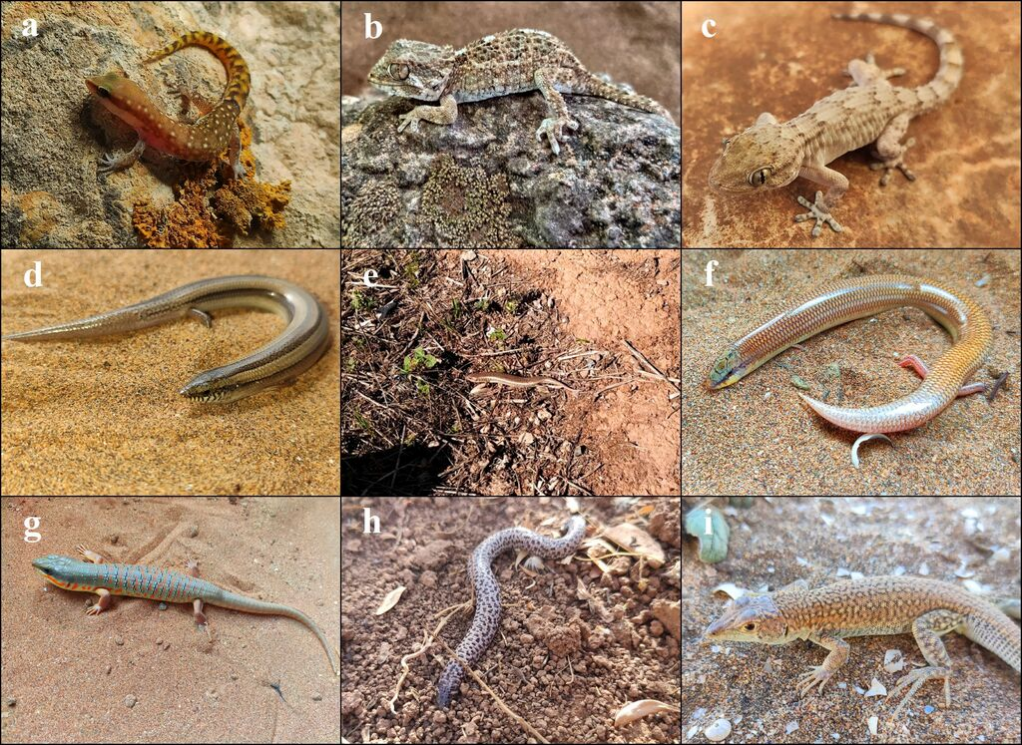
Species of Squamates present in Souss-Massa National Park. **a** *Saurodactylusbrosseti* complex, photo by A. Elbahi; **b** *Tarentolachazaliae*, photo by A. Elbahi; **c** *Tarentolamauritanica*, photo by A. Elbahi; **d** *Chalcidesmionecton*, photo by A. Elbahi; **e** *Chalcidespolylepis*, photo by A. Elbahi; **f** *Chalcidessphenopsiformis*, photo by A. Elbahi; **g** *Eumecesalgeriensis*, photo by A. Elbahi; **h** *Trogonophiswiegmanni*, photo by A. Elbahi; **i** *Acanthodactylusaureus*, photo by A. Elbahi.

*Saurodactylusbrosseti* complex (Fig. 4a)

**Common name**: Morocco lizard-fingered gecko

**IUCN status**: Least Concern

**Comment**: *Saurodactylusbrosseti* is a species endemic to Morocco; it was previously considered a subspecies of *Saurodactylusmauritanicus* and then raised to species level based on morphology (Bons and Geniez 1996). Later, genetic analysis supported the recognition of *S.brosseti* as a full species (Rato and Harris 2008). Further studies on the evolutionary and biogeographical history of the *S.brosseti* complex uncovered four highly divergent and allopatric mitochondrial lineages; North, Anti-Atlas, East and South lineages (Rosado et al. 2017). Recently, Javanmardi et al. (2019) divided *S.brosseti* complex into five species, based on morphological and phylogenetic analysis combined with genetic data provided by Rosado et al. (2017); *S.brosseti* (North lineage), *S.elmoudenii* (Anti-Atlas lineage), *S.harrisii* (South lineage), *S.slimanii* (East lineage) and *S.splendidus* (Southeast lineage). It is important to note that the North lineage occurs from some kilometres south of Agadir to its most northern known geographic location. The South lineage occurs from Agadir to the coast of the Atlantic Sahara in the south. This means that the region around Agadir (including SMNP) can be considered as a contact zone between both Northern and Southern lineages, which might result in some shared colour morphs (due to limited genetic exchange (Javanmardi et al. 2019)). However, genetic analyses are needed to determine which lineage SMNP individuals belong to and confirm whether this National Park is a contact zone between both lineages or if it only hosts a single lineage.

**Family Phyllodactylidae**

*Tarentolachazaliae* (Fig. 4b)

**Common name**: Helmethead Gecko

**IUCN status**: Vulnerable

**Comment**: Its common name, Helmethead gecko, is derived from the shape of its head, which is covered with small granulations and long pointed occipital tubercles that gives it the appearance of a helmet. This made it very distinct from other species of the *Tarentola* genus. This species is a member of the Phyllodactylidae family, endemic to the north-western Atlantic coast of Africa, from the region of Agadir in Morocco to Senegal. Morocco alone has around three-quarters of the global *T.chazaliae* distribution (Bons and Geniez 1996). This species usually lives close to the littoral zone, where the humidity is high. However, it can be found at Sidi Ahmed Laaroussi near Smara, 144 km from the ocean (Sánchez-Vialas and Aznar-González de Rueda 2016). In SMNP and during daytime, *T.chazaliae* was observed mostly inactive under rocks (or under plastic waste). In contrast, it was seen active with round and fully opened pupils during nocturnal surveys. Individuals showed a terrestrial behaviour most of the time.

*Tarentolamauritanica* (Fig. 4c)

**Common name**: Moorish Gecko

**IUCN status**: Least Concern

**Comment**: *Tarentolamauritanica* has a large distribution range, mainly across the coastal Mediterranean Regions (Schleich et al. 1996). According to Geniez et al. (1999), three morphologically distinct subspecies are present in Morocco; *T.m.juliae*, *T.m.mauritanica* and *T.m.pallida*. However, Rato et al. (2016) recovered four clades of *T.mauritanica* in Morocco; the Europe/North Africa clade, the Maghreb/South Iberia clade, the Central Morocco clade and the Central/Southern Morocco clade. In SMNP, *T.mauritanica* is represented by the Central/Southern Morocco clade and it has the widest distribution range of all SMNP geckos (it was present at 18 sites, which corresponded to 60% of all surveyed sites). During nocturnal surveys, this species was observed predating on nocturnal insects near light sources and street lamps around villages. Despite being chiefly nocturnal or crepuscular, it was also seen active during the day on rocks, tree trunks, stone walls and buildings. Males were larger compared to females with broader heads.

**Family Scincidae**

*Chalcidesmionecton* (Fig. 4d)

**Common name**: Mionecton Skink

**IUCN status**: Least Concern

**Comment**: A species endemic to Morocco, represented by two subspecies highly supported by phylogenetic analysis (Carranza et al. 2008); *C.m.mionecton*, which is distributed from the Atlantic coast of Tangier to Cap Rhir (it can also be found in some few valleys of the western High Atlas) and *C.m.trifasciatus*, which is distributed from Cap Rhir to Labyar and it reached the lower Souss valley (Martínez del Mármol et al. 2019). Unlike the first subspecies that generally has only four digits, the *C.m.trifasciatus* subspecies is characterised by having five digits (Martínez del Mármol et al. 2019). The latter occurs in SMNP, mostly in sandy areas with a loose substrate and it can be found by lifting flat rocks. When they sense a potential danger or feel disturbed, they disappear instantly deep into the sandy soils. This species was rarely seen moving above the ground.

*Chalcidespolylepis* (Fig. 4e)

**Common name**: Many-scaled Skink

**IUCN status**: Least Concern

**Comment**: *Chalcidespolylepis* was first described as a subspecies of *Chalcidesocellatus*, then raised to species level according to morphological analysis (Lanza 1957), which was later supported by phylogenetic analysis (Carranza et al. 2008). This relatively large skink is endemic to the western part of Morocco and it can be distinguished from other species of the *Chalcides* genus by the high number of scales around the body (between 34 and 40 rows of dorsal scales at mid-body) (Bons and Geniez 1996). This species is considered a rare species in the present work as it was only found once at site 18 in a small rocky area near the Massa River during the late summer of 2020.

*Chalcidessphenopsiformis* (Fig. 4f)

**Common name**: Duméril's Wedge-snouted Skink

**IUCN status**: Least Concern

**Comment**: Previously included within the genus *Sphenops* and considered later as a member of the *Chalcides* genus after morphological and phylogenetic analysis (Carranza et al. 2008). This species is distributed in the north-western African coast, from Agadir (Morocco) to Senegal through to the Mauritanian coast (Bons and Geniez 1996). So far, no subspecies have been described. In SMNP, this skink was the most abundant species of the Scincidae family with the widest distribution range. It is well adapted to living and moving under the sand and characterised by reduced limbs that help with undulatory “swimming” motion in sand. This species was only observed active a few times during nocturnal surveys and, once approached, specimens dived instantly deep into the sandy substrate to hide. However, it was never sighted moving above the ground during diurnal surveys and, in order to find it, we followed its tracks which form long regular undulations on the sand.

*Eumecesalgeriensis* (Fig. 4g)

**Common name**: Algerian Skink

**IUCN status**: Least Concern

**Comment**: *Eumecesalgeriensis* is a large skink characterised by a massive oval head slightly distinct from the neck and by a dorsal pattern with orange-reddish spots. This species can be found in both Algeria and Morocco. The species comprises two subspecies; *E.a.algeriensis*, which has the widest distribution range in Morocco compared to *E.a.meridionalis*, which can be found in the eastern part of Morocco and in the north-west of Algeria (Sindaco and Jeremcenko 2008, Martínez del Mármol et al. 2019). This species is represented in SMNP by the subspecies *E.a.algeriensis* and was only encountered twice at a single site (site 18) between dense bush vegetation.

**Family Trogonophiidae**

*Trogonophiswiegmanni* (Fig. 4h)

**Common name**: Checkerboard Worm Lizard

**IUCN status**: Least Concern

**Comment**: This worm lizard is monotypic within the genus *Trogonophis* and is the only representative of the Trogonophiidae family in North Africa. It is endemic to the Maghreb Region and is present in Morocco, Algeria and Tunisia. Based on colouration, two subspecies were described (Bons and Geniez 1996) and both are present in Morocco; *T.w.wiegmanni*, with a pale yellow ground colour and *T.w.elegans* with light pink or light mauve ground colour (endemic to the country). Phylogenetic and molecular studies revealed three lineages in the Maghreb (Salvi et al. 2018); *elegans* lineage corresponds to *T.w.elegans*, while western *wiegmanni* lineage (occurs in eastern Morocco) and eastern *wiegmanni* lineage (occurs in Algeria and Tunisia) include *T.w.wiegmanni*. The Checkerboard Worm Lizard is represented in SMNP by the endemic subspecies *T.w.elegans.* It is considered a rare species in the present work since it was only found twice with very restricted distribution (sites 24 and 25). Both individuals were found under rocks in *Euphorbia* steppe. However, this fossorial species may be more common than our records indicate because it spends most time in burrows or under rocks and rarely appears above the ground.

**Family Lacertidae**

*Acanthodactylusaureus* (Fig. 4i)

**Common name**: Golden Fringe-fingered Lizard

**IUCN status**: Least Concern

**Comment**: *Acanthodactylusaureus* occurs in the African Atlantic coast from Agadir (Morocco) to Mauritania, with some records in Senegal (Velo-Antón et al. 2018). So far, no subspecies have been described. *A.aureus* was the most frequently encountered reptile species during the study period; this species alone represented 41.36% of all TCVES observations and was present at 17 sites (representing 56.67% of all surveyed sites). It was active during daytime throughout the year, even during overcast and cool weather. It was mainly observed basking not far from its burrows, hunting insects and escaping from predators, such as snakes and the Northern Bald Ibis. Once encountered, it runs instantly and hides inside burrows or bushes. Males can be easily differentiated by their visible hemipenal bulges. During the breeding season, males are characterised by a golden yellow colour, while females are brown with relatively visible lines of white dots.

**Figure 5.**
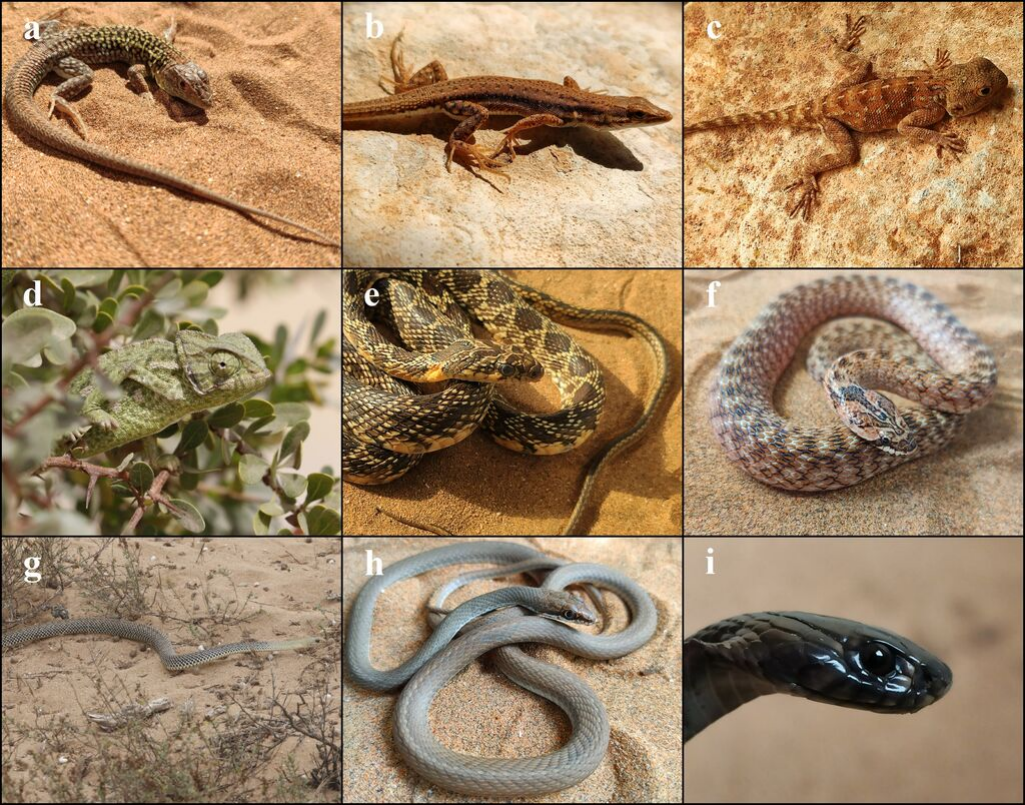
Species of Squamates present in Souss-Massa National Park. **a** *Acanthodactylusmargaritae*, photo by A. Elbahi; **b** *Mesalinaolivieri*, photo by A. Elbahi; **c** *Agamaimpalearis*, photo by A. Elbahi; **d** *Chamaeleochamaeleon*, photo by W. Oubrou; **e** *Hemorrhoishippocrepis*, photo by A. Elbahi; **f** *Macroprotodonbrevis*, photo by A. Elbahi; **g** *Malpolonmonspessulanus*, photo by W. Oubrou; **h** *Psammophisschokari*, photo by A. Elbahi; **i** *Najahaje*, photo by A. Elbahi.

*Acanthodactylusmargaritae* (Fig. 5a)

**Common name**: Margarita's Fringe-fingered Lizard

**IUCN status**: Not evaluated

**Comment**: A newly-described species, first considered as *Acanthodactylusbusacki* within the *Acanthodactyluspardalis* species-group. It was later raised to species rank (Tamar et al. 2017), based on comparative genetic and morphological analyses, which revealed two genetically divergent lineages within *A.busacki*; the Northern lineage was described as the new species *A.margaritae*, distinguished by its weakly-keeled dorsal scales and its characteristic colour pattern, while the Southern lineage included the nominal species *A.busacki*. *A.margaritae* is endemic to Morocco and can be found between the High Atlas and Anti-Atlas Mountains; from around Tamri in the north to Tiznit surroundings in the south and the Atlantic coast in the west to the Souss Valley in the east (Tamar et al. 2017). It is a widespread and common species in SMNP and it was present at 25 sites (83.33% of all surveyed sites). It was observed active during daytime throughout the year and it can also be seen active during overcast or cool weather, although less abundant compared to sunny days. It spends most of the time basking, capturing prey and escaping from any potential danger by running and hiding in burrows or under bushes. This species is characterised by a yellowish colouration during the breeding season, while it weakens or disappears later in summer. *A.margaritae* was often observed sympatric with *A.aureus* at many sites along the SMNP sandy coast.

*Mesalinaolivieri* (Fig. 5b)

**Common name**: Olivier's Small Lizard

**IUCN status**: Least Concern

**Comment**: *Mesalinaolivieri* is widely distributed across North Africa from the Atlantic Sahara towards Egypt, Jordan, Israel, southern Iraq and northern Saudi Arabia (Martínez del Mármol et al. 2019). Kapli et al. (2015) suggest that the *Mesalinaolivieri* complex may be of African origin, unlike most species of *Mesalina* genus that originated in Arabia or the Middle East. *M.olivieri* was rarely encountered in SMNP and was restricted to site 23. This species had the lowest observed abundance of all Lacertidae representatives in this National Park. Only four individuals were observed in rocky open ground with scarce vegetation formed by *Launaeaarborescens*.

**Family Agamidae**

*Agamaimpalearis* (Fig. 5c)

**Common name**: Bibron's Agama

**IUCN status**: Least Concern

**Comment**: A species endemic to the Maghreb, where it can found in Morocco, Algeria and northern Mauritania (Martínez del Mármol et al. 2019). Brown et al. (2002) identified two lineages within *A.impalearis* in Morocco, based on mitochondrial DNA analyses; the first lineage occurs north and west of the Atlas Mountains (NW lineage) while the second occurs south and east of these mountains (SE lineage). However, the difference between these two lineages turned out to be less than between lineages of other species of genus *Agama* (Gonçalves et al. 2012). In SMNP, *A.impalearis* was usually observed in the rocky steppe of *Euphorbia*, where the substrates constitute a favourable habitat. Individuals were active during daytime and were seen capturing insects and basking on the top of rock piles, stone walls near villages, logs and plants of *Euphorbia*. Adults engage in the territorial defence of their home range against other members of their species. When threatened, they run instantly and hide in rock fissures, rodents’ burrows or under thick vegetation such as *Euphorbiaechinus*. During winter, individuals demonstrated reduced activity and were usually found inactive under rocks.

**Family Chamaeleonidae**

*Chamaeleochamaeleon* (Fig. 5d)

**Common name**: Common Chameleon

**IUCN status**: Least Concern

**Comment**: The common chameleon is the only species of the Chamaeleonidae family found in the Maghreb Region, represented by the nominal subspecies *C.c.chamaeleon* (Basso et al. 2019). However, individuals from the Maghreb can be genetically differentiated and two Mediterranean haplotypes occur in this Region (Dimaki et al. 2008); the western Mediterranean haplotypes that occur in Morocco and the eastern Mediterranean haplotypes that can be found in Tunisia. The common chameleon was mainly observed during diurnal surveys in areas with dense vegetation, while it was only spotted once during nocturnal surveys (observed in October). Despite being an arboreal reptile, this species was once found taking shelter in rock piles at site 26 after it walked a few metres on the sandy substrate. Males are relatively smaller than females and can be distinguished by their hemipenal pockets, a relatively longer tail and the greater height of their helmet.

**Family Colubridae**

*Hemorrhoishippocrepis* (Fig. 5e)

**Common name**: Horseshoe Whip Snake

**IUCN status**: Least Concern

**Comment**: The Horseshoe Whip Snake occurs in the Iberian Peninsula, the Maghreb Region (more precisely, Morocco, Algeria and Tunisia) and the Mediterranean Islands of Pantelleria and Sardinia (Martínez del Mármol et al. 2019). Two subspecies can be found; *H.h.hippocrepis*, which occurs in most of the distribution range of the species, while *H.h.nigrescens* is restricted to the Pantelleria Island (Carranza et al. 2006). This non-venomous snake was the most encountered species of snakes in SMNP during the period 2019 to 2020 (representing 28.21% of all observed snakes, based on TCVES data). It was found at different sites, either in natural habitats or near human environments (hedges, stone walls, ruins and farms). The head of this snake is well differentiated from the body and bears a characteristic horseshoe pattern.

*Macroprotodonbrevis* (Fig. 5f)

**Common name**: Western False Smooth Snake

**IUCN status**: Near Threatened

**Comment**: This species was previously considered as *Macroprotodoncucullatus*, the sole representative of the monospecific genus *Macroprotodon*. Then, it was elevated to species level, based on morphological and genetic analyses (Wade 2001, Carranza et al. 2004), which showed that *M.brevis* is distinct from *M. cucullatus. M.brevis* is distributed across the Iberian Peninsula, Algeria and Morocco. The latter hosts three subspecies (Martínez del Mármol et al. 2019); *M.b.brevis*, *M.b.ibericus* and *M.b.textilis*. This species is the smallest snake found in SMNP (none of the observed individuals exceeded 50 cm in total length) and represented by the endemic subspecies *M.b.brevis.* Ten individuals were observed; nine were found inactive under rocks (sometimes burrowed in the sand under large rocks) during diurnals surveys, while a single individual was found on the surface during a nocturnal survey. At site 19, variation amongst the same population can be observed and some individuals had different patterns and background colour of the head and body. This species is a mildly venomous rear-fanged snake, yet it did not show aggressive behaviour nor attempt to bite humans while being handled.

**Family Lamprophiidae**

*Malpolonmonspessulanus* (Fig. 5g)

**Common name**: Western Montpellier Snake

**IUCN status**: Least Concern

**Comment**: This species can be found in the Iberian Peninsula, France, Italy (restricted to Liguria), Morocco and Algeria (Carranza et al. 2006, Mangiacotti et al. 2014). Two subspecies occur in Morocco; the nominal subspecies *M.m.monspessulanus* and *M.m.saharatlanticus*. The Souss Massa Region can be considered as a contact zone between both subspecies. Intermediate specimens have already been found in the coastal area between Agadir to Tiznit and in the lower Souss Valley (Geniez et al. 2006). This species was observed during daytime, mostly in the sandy coastal steppe of SMNP. It has a much-reduced activity during the coldest months and we could not find any individual during the winters of both 2019 and 2020. It has the most remarkable sexual dimorphism of all SMNP snakes; males were larger and had a relatively longer head than females, their supralabial scales were characterised by a pale greenish or greyish colouration, unlike females that had a brown or russet-red colouration. *M.monspessulanus* is a mildly venomous rear-fanged snake and cases of human envenomation by this species are rare.

*Psammophisschokari* (Fig. 5h)

**Common name**: Schokari Sand Racer

**IUCN status**: Not evaluated

**Comment**: This common colubrid has a wide distribution range and it occurs in North Africa, Middle East, Afghanistan, Pakistan and India (Martínez del Mármol et al. 2019). It is widely distributed across Morocco and can be found in different types of habitats. In general, three phenotypes can be found in the country (Bons and Geniez 1996); a striped morph, a unicoloured morph and a slightly striped morph (sometimes indistinct) with a series of dark dots along the dorsolateral area. However, these morphs are closely related and belong to the same Moroccan clade (Rato et al. 2007) and, therefore, the colour pattern of the three morphs does not reflect genetic variability. Colour variations might be the result of an ecological adaptation to the environment (Rato et al. 2007). During the study period, four individuals were observed and all of them were uniformly brown; one adult specimen was found inactive under a rock (during mid-autumn), while the others were active. Once encountered, they moved fast on the sandy substrate to take cover in the bushes. Despite being a rear-fanged snake, *P.schokari* does not attempt to bite humans while being handled and cases of envenomation by this snake are exceptional (a case of human envenomation has been documented in the Sultanate of Oman) (Ineich et al. 2020).

**Family Elapidae**

*Najahaje* (Fig. 5i)

**Common name**: Egyptian Cobra

**IUCN status**: Least Concern

**Comment**: *Najahaje* has a wide distribution, ranging across North Africa, the Sahel Region, central and eastern Africa (Martínez del Mármol et al. 2019). This species is the second longest snake and the only representative of the Elapidae family in Morocco. During the study period, all observations were restricted to the southern part of SMNP, from the sandy coastal steppe to the rocky *Euphorbia* steppe. However, during a few surveys outside the National Park, we observed this species in the High Atlas Mountains only a few kilometres away from the northern limit of SMNP. Despite being mainly a nocturnal species, one adult and one juvenile were spotted active during overcast days; the first one was observed during late summer while the other one was seen active during early autumn. Juveniles were uniformly black on the anterior third of the body (including the ventral area), while the rest of the body had a yellow colouration covered with black and brown spots. Adult individuals were mostly characterised by a uniform black colour. *N.haje* is a highly venomous species (neurotoxic venom) and regularly causes human fatalities.

**Family Viperidae**

*Daboiamauritanica*

**Common name**: Moorish Viper

**IUCN status**: Near Threatened

**Comment**: *Daboiamauritanica* is a viper endemic to the Maghreb Region and includes seven different lineages according to recent genetic analysis (Martínez-Freiría et al. 2017); six of these lineages are endemic to Morocco. This species is the longest viper in Morocco and has the widest distribution. It is the only representative of the Viperidae family in SMNP and it is considered a rare species in the present work (only observed once). During the early autumn of the year 2019 and just after an unusually heavy downpour, a single adult individual was found dead in the northern part of the National Park (site 1). It had been killed by locals probably after it moved from Oued Souss towards agricultural fields. *D.mauritanica* is a highly venomous snake and many cases of envenomation have been reported in Morocco. Most envenomation cases in northern Morocco are caused by this viper (Argaz et al. 2013), probably due to its high abundance and its wide distribution compared to other Moroccan vipers.

## Discussion

In the past decades, human activities, such as overexploitation of natural resources, clearing land for agriculture or tourism purposes and overgrazing, have become increasingly prevalent, putting significant pressure on the Moroccan biodiversity. In the coming decades, it is predicted that climate change, which is influenced by human activities (Trenberth 2018), will negatively affect the Moroccan endemic reptile richness and cause substantial reductions in species-rich areas (Martínez-Freiría et al. 2013). Future reductions in suitable areas were predicted for half of the Moroccan endemic reptile species, amongst which four species might become highly vulnerable to extinction (Martínez-Freiría et al. 2013). The conservation of individual species can be mainly achieved by protecting their habitats and, therefore, establishing protected areas can be considered a promising strategy to mitigate biodiversity declines and habitat destruction in Morocco. SMNP has been primarily created to maintain and protect its terrestrial and marine biodiversity and ecosystems. Reptiles, which remain insufficiently studied in this National Park, benefit from the legal management of the diverse ecosystems and habitats. This protection will be essential in order to expand our knowledge of reptiles and their ecological importance within the confines of the National Park. SMNP is, however, divided into different zones; some of which are fully protected (all human activities are strictly limited or prohibited), such as Reserves, while other zones receive less or almost no protection. The efficiency of reptile biodiversity protection in these less protected zones has been poorly studied and needs to be assessed.

The current reptile inventory brings the total number of reptile species in SMNP to 23 species, using two combined techniques. In the present work, new records of one species of sea turtle (*Dermochelyscoriacea*) and two species of medically significant snakes (*Najahaje* and *Daboiamauritanica*) were documented for the first time in the study area. Based on TCVES data, accumulation curves showed that the current inventory of SMNP reptiles is nearly complete. The non-parametric estimators predicted a total richness ranging from 22.74 to 25 species, which means that the 22 species found using the TCVES technique represent between 88% and 96.74% of the total estimated species. These findings suggest that 3.26% to 12% more species are expected to be recorded in order to achieve the asymptote and completeness of the SMNP reptile inventory. However, the TCVES technique yielded an excellent representation of reptile species present during the period from the 8 January 2019 to the 5 December 2020.

The main novelty since the previously established reptile checklist by Mellado et al. (1988) was the description of new species, some of which resulted from the systematic revision of other species or subspecies, while other species were assigned to new genera. Mellado et al. (1988) provided a checklist with a total of 22 species. These latter were previously presented as follows: *Testudograeca, Mauremys Leprosa, Dermochelyscoriacea, Trogonophiswiegmanni, Saurodactylusmauritanicus, Geckonia chazaliae, Tarentolamauritanica, Agama bibronii, Chamaeleochamaeleon, Chalcidesmionecton, Chalcidesocellatus, Eumecesalgeriensis, Sphenops sphenopsiformis, Acanthodactyluserythrurus, Acanthodactylusaureus, Acanthodactyluspardalis, Mesalinaolivieri, Malpolonmonspessulanus, Coluberhippocrepis, Macroprotodoncucullatus, Natrixmaura* and *Psammophisschokari.* However, six species within this previous checklist have either changed their genus or experienced systematic modifications, which led to the description of new species, while three species were absent in our new inventory. *Geckonia chazaliae, Sphenops sphenopsiformis* and *Coluberhippocrepis* are now included within the genera *Tarentola, Chalcides* and *Hemorrhois*, respectively (Carranza et al. 2002, Carranza et al. 2006, Carranza et al. 2008). According to genetic and morphological analyses, *Saurodactylusmauritanicus, Acanthodactyluspardalis* and *Macroprotodoncucullatus* have been revised to new species descriptions and, in the region including SMNP, are represented by *Saurodactylusbrosseti* complex, *Acanthodactylusmargaritae* and *Macroprotodonbrevis*, respectively (Bons and Geniez 1996, Wade 2001, Carranza et al. 2004, Rato and Harris 2008, Rosado et al. 2017, Tamar et al. 2017).

*Chalcidesocellatus, Acanthodactyluserythrurus* and *Natrixmaura* were recorded in the previous checklist. However, none of them was found over the course of our study. Mellado et al. (1988) observed some relatively large individuals of *Chalcides* along Oued Massa without capturing them. They noted that these individuals seemed more similar to *C.ocellatus* than *C.polylepis* and, therefore, they provisionally considered all individuals as *C.ocellatus*. However, our observed individual from Oued Massa in SMNP was clearly a *C.polylepis* and, therefore, we believe that this species was misidentified in the previous work and was treated as *C.ocellatus*. *Acanthodactyluserythrurus* was not observed during our study period; neither inside SMNP nor in its vicinity. This might be explained by either a reduction of *A.erythrurus* distribution range (in the past, it was very common north and west of the Atlas Mountains and in these mountains (Martínez del Mármol et al. 2019)) or by species misidentification (females and juveniles of *A.erythruruslineomaculatus* resemble those of *A.margaritae* (Martínez del Mármol et al. 2019)). *Natrixmaura* was also absent during our survey. However, we found it near Youssef Ibn Tachfine Dam that was built on Oued Massa just a few kilometres from SMNP. Mellado et al. (1988) found *N.maura* near freshwater bodies, most likely when they conducted a survey near Oued Massa bridge (national road 1); *N.maura* was later observed in the same area by other researchers. This area was previously included within the limits of MNPP, which was supposed to cover 48,000 hectares, compared to 33,800 hectares for the actual National Park. SMNP includes now reduced freshwater bodies and a lower abundance of amphibians (considered important prey for *N.maura*) compared to the former MNPP and, therefore, a lower chance of encountering *N.maura*.

Despite its small surface area, SMNP contains nearly 20% of all known Moroccan terrestrial and marine reptiles (116 species (Bouazza et al. 2021)), including four endemic species. Ten National Parks have been created in Morocco and three of them can be defined as coastal; Souss-Massa National Park (SMNP), Al Hoceima National Park (AHNP) and Khnifiss National Park (KNP). Based on the reptile distribution maps in northern Morocco (Mediani et al. 2015), approximately 18 to 20 reptile species can be found in the AHNP and its vicinity, while approximately 20 reptile species were recorded in the KNP (Qninba 2013). This means that SMNP has the richest reptilian fauna amongst all Moroccan coastal National Parks. Its reptilian fauna is also richer than many other coastal National Parks in the Maghreb and Mediterranean Regions, such as Circeo National Park in Italy (16 reptile species (Vignoli et al. 2017)), Port-Cros National Park in France (17 reptile species (Marchand et al. 2019)), Gouraya National Park in Algeria (19 herpetofauna species (Boumaour et al. 2018)) and Banc d'Arguin National Park in Mauritania (21 reptile species (Sow et al. 2014)). This makes SMNP an important biodiversity hotspot for reptile species in the Mediterranean Region. However, it is possible that the use of different reptilian biodiversity assessment methods could generate differences in the number of species found. Therefore, some of the species richness differences observed between the current study and similar studies might be partially explained by methodological differences.

In terms of conservation concern, five species amongst the SMNP reptiles are classified as “vulnerable” on the IUCN Red List (IUCN 2021), while two species are classified as “near threatened”. All these threatened/near-threatened species are experiencing a continuous population size decline in their total distribution range and are under several threats (IUCN 2021). Habitat loss and fragmentation are common threats to these reptile species. Vulnerable species, such as *Tarentolachazaliae* and *Testudograeca*, are being collected for the international pet trade, while species of ophidians are generally killed when they are encountered or taken from their natural habitats and used for snake-charming performances. Meanwhile, vulnerable marine reptiles (*Carettacaretta* and *Dermochelyscoriacea*) and their habitats are mainly threatened by fishery bycatch, pollution, coastal development, pathogens and climate change (Wallace et al. 2010). In the face of the increasing threats, precautionary conservation measures need to be taken in order to protect the remaining reptile biodiversity.

## Figures and Tables

**Table 1.** List of reptile species within Souss-Massa National Park. TCVES = time-constrained visual encounter surveys, AO = additional observations.

**No**	**Taxa**	**Survey sites**	**Technique**
	**Order Chelonii**		
	**Family Testudinidae**		
***1***	*Testudograeca*	1, 2, 3, 5, 6, 7, 8, 9, 10, 11, 12, 13, 15, 16, 17, 18, 19, 22, 23, 24, 25, 26, 27, 28, 29 and 30	TCVES
	**Family Geoemydidae**		
***2***	*Mauremysleprosa*	18	TCVES
	**Family Cheloniidae**		
***3***	*Carettacaretta*	2, 8, 13, 19 and 22	TCVES, AO
	**Family Dermochelyidae**		
***4***	*Dermochelyscoriacea*	8, 13 and 30	AO
	**Order Squamata**		
	**Family Sphaerodactylidae**		
***5***	*Saurodactylusbrosseti* complex	5, 17, 18, 23, 24, 25, 26, 27 and 30	TCVES
	**Family Phyllodactylidae**		
***6***	*Tarentolachazaliae*	5, 12, 13, 15, 16, 17, 19, 22, 25, 26, 27, 28 and 29	TCVES, AO
***7***	*Tarentolamauritanica*	1, 3, 4, 5, 6, 7, 8, 9, 10, 11, 13, 15, 16, 18, 21, 23, 29 and 30	TCVES, AO
	**Family Scincidae**		
***8***	*Chalcidesmionecton*	1, 3, 5, 6, 9, 11, 13, 15, 16, 18, 21, 23, 26, 27, 29 and 30	TCVES, AO
***9***	*Chalcidespolylepis*	18	TCVES
***10***	*Chalcidessphenopsiformis*	2, 3, 5, 7, 8, 9, 10, 11, 12, 13, 15, 16, 18, 19, 20, 21, 22, 26, 28, 29 and 30	TCVES
***11***	*Eumecesalgeriensis*	18	TCVES
	**Family Trogonophiidae**		
***12***	*Trogonophiswiegmanni*	24 and 25	TCVES
	**Family Lacertidae**		
***13***	*Acanthodactylusaureus*	2, 7, 8, 9, 10, 11, 12, 13, 15, 16, 19, 20, 21, 22, 28, 29 and 30	TCVES
***14***	*Acanthodactylusmargaritae*	1, 3, 5, 6, 7, 8, 9, 10, 11, 12, 13, 14, 15, 16, 18, 19, 20, 21, 22, 23, 26, 27, 28, 29 and 30	TCVES
***15***	*Mesalinaolivieri*	23	TCVES
	**Family Agamidae**		
***16***	*Agamaimpalearis*	2, 3, 5, 6, 7, 17, 23, 24, 25 and 27	TCVES
	**Family Chamaeleonidae**		
***17***	*Chamaeleochamaeleon*	2, 3, 5, 6, 7, 10, 11, 12, 13, 15, 16, 18, 26 and 28	TCVES, AO
	**Family Colubridae**		
***18***	*Hemorrhoishippocrepis*	1, 3, 5, 6, 7, 9, 11, 13, 15, 16, 18, 19 and 30	TCVES, AO
***19***	*Macroprotodonbrevis*	5, 12, 13, 15, 16, 18, 19, 28 and 29	TCVES, AO
	**Family Lamprophiidae**		
***20***	*Malpolonmonspessulanus*	3, 5, 7, 10, 11, 12, 13, 15, 16, 17, 18, 19 and 22	TCVES, AO
***21***	*Psammophisschokari*	5, 7, 16, 23, 26, 29 and 30	TCVES, AO
	**Family Elapidae**		
***22***	*Najahaje*	23, 26, 27 and 28	TCVES, AO
	**Family Viperidae**		
***23***	*Daboiamauritanica*	1	TCVES
